# Qualitative observation instrument to measure the quality of parent-child interactions in young children with type 1 diabetes mellitus

**DOI:** 10.1186/1471-2431-14-145

**Published:** 2014-06-10

**Authors:** Anke Nieuwesteeg, Esther Hartman, Frans Pouwer, Wilco Emons, Henk-Jan Aanstoot, Edgar Van Mil, Hedwig Van Bakel

**Affiliations:** 1 Center of Research on Psychology in Somatic diseases (CoRPS), Department of Medical and Clinical Psychology, Tilburg University, PO Box 90153, Tilburg LE 5000, The Netherlands; 2 Department of Methodology and Statistics, Tilburg University, PO Box 90153, Tilburg LE 5000, The Netherlands; 3 Diabeter, Rotterdam TG 3011, The Netherlands; 4 Kidz&Ko, Jeroen Bosch Hospital, P.O. Box 90153, ’s-Hertogenbosch 5200 ME, The Netherlands; 5 Department of TRANZO, Scientific Center for Care and Welfare, Tilburg University, PO Box 90153, Tilburg LE 5000, The Netherlands

**Keywords:** Type 1 diabetes mellitus, Parent–child interaction, Behavior, Children, Parents, Mealtime, Rating scale

## Abstract

**Background:**

In young children with type 1 diabetes mellitus (T1DM), parents have complete responsibility for the diabetes-management. In toddlers and (pre)schoolers, the tasks needed to achieve optimal blood glucose control may interfere with normal developmental processes and could negatively affect the quality of parent–child interaction. Several observational instruments are available to measure the quality of the parent–child interaction. However, no observational instrument for diabetes-specific situations is available. Therefore, the aim of the present study was to develop a qualitative observation instrument, to be able to assess parent–child interaction during diabetes-specific situations.

**Methods:**

First, in a pilot study (n = 15), the observation instrument was developed in four steps: (a) defining relevant diabetes-specific situations; (b) videotaping these situations; (c) describing all behaviors in a qualitative observation instrument; (d) evaluating usability and reliability. Next, we examined preliminary validity (total n = 77) by testing hypotheses about correlations between the observation instrument for diabetes-specific situations, a generic observation instrument and a behavioral questionnaire.

**Results:**

The observation instrument to assess parent–child interaction during diabetes-specific situations, which consists of ten domains: “emotional involvement”, “limit setting”, “respect for autonomy”, “quality of instruction”, “negative behavior”, “avoidance”, “cooperative behavior”, “child’s response to injection”, “emphasis on diabetes”, and “mealtime structure”, was developed for use during a mealtime situation (including glucose monitoring and insulin administration).

**Conclusions:**

The present study showed encouraging indications for the usability and inter-rater reliability (weighted kappa was 0.73) of the qualitative observation instrument. Furthermore, promising indications for the preliminary validity of the observation instrument for diabetes-specific situations were found (r ranged between |.24| and |.45| for significant correlations and between |.10| and |.23| for non-significant trends). This observation instrument could be used in future research to (a) test whether parent–child interactions are associated with outcomes (like HbA_1c_ levels and psychosocial functioning), and (b) evaluate interventions, aimed at optimizing the quality of parent–child interactions in families with a young child with T1DM.

## Background

When young children are diagnosed with type 1 diabetes mellitus (T1DM), parents have to take complete responsibility for the daily diabetes-management of their child, 24 hours a day, 7 days a week. Several times a day, they have to monitor the blood glucose level, administer insulin, regulate food intake, and guard these parameters in conjunction with the level of physical activity of their child with diabetes. The tasks needed to achieve optimal blood glucose control, however, may interfere with normal and age appropriate behaviors that occur in the toddler and pre-school years (e.g., increase in autonomy, independence-seeking, refusing food, or oppositional behavior) [1]. This may also affect the quality of the parent–child interaction [2]. If the quality of the parent–child interaction is non-optimal, this might also have a negative impact on the diabetes self-care behaviors of parents and children.

Given the importance of this topic, it is surprising that the number of studies examining the quality of the parent–child interaction in families with young children with T1DM is limited [3]. Few studies have examined the quality of parent–child interaction in children with T1DM, and existing studies mainly focused on older children with T1DM (>8 years) [4-8] or used a wide age range, from 1–14 years [9,10]. Moreover, most studies mainly used self-report measures or semi-structured interviews [4,5,7,9-11]. When investigating the quality of parent–child interaction, videotaped interactions and rating scales may give more detailed information about the various aspects of parent–child interactions [12,13]. Moreover, self-report measures and interviews by definition reflect a subjective quantification of concepts from the perspective of parents whereas observed interactions by an independent observer can provide more objective data [3].

As far as we know, only two research groups were identified that studied the quality of parent–child interaction in young children with T1DM using an observation method [1,14-18]. These studies observed behaviors of both parents and children during mealtime, which were counted by frequency (e.g., how often the child was encouraged by the parents to keep eating), time intervals (e.g., see if the child is eating on second 10, second 20 etc.) or whether a specific behavior was present or not during the observation. However, counting the number of specific behaviors and using time intervals have some disadvantages. No information about the affective quality of the dyadic behavior is reflected in the observed behaviors. In contrast, applying rating scales has the advantage that affective components can be taken into account. This way of coding observational data allows making many dimensions and subtle differences in behaviors. Moreover, the predictive value of rating scales has proved to be more accurate than just counting specific behaviors [19-21]. Furthermore, when using rating scales, the behaviors of the parents can be evaluated in the context of the behaviors of the child [20], which is important when observing the quality of parent–child interaction. An additional advantage of rating scales is that it is more time efficient [12], and can cost up to five times less time than counting all behaviors [19]. Hence, the use of rating scales in observational studies gives a clinical picture which results in more specific implications for intervention purposes and is time efficient [3].

In the past decades, several generic rating scales have been developed for assessing different aspects of the quality of parent–child interactions for use in the general population [3], e.g., the Emotional Availability Scales (EAS) [22], scales developed by the National Institute of Child Health and Human Development (NICHD) [23], and scales developed by Erickson, Sroufe and Egeland [24]. These rating scales were designed to cover various aspects of parent–child interaction irrespective of an underlying disease. Moreover, these tools were not specifically designed to use in a clinical sample and did not take into account disease-specific behaviors such as the parent’s and child’s reaction to medical tasks or the emphasis of the disease during the interaction. However, disease-specific observational rating scales are still not available.

The aim of this study is therefore to develop such an observation instrument for diabetes-specific situations to assess the quality of parent–child interactions in young children (0–7 years) with T1DM by means of direct observations. Furthermore, the usability, inter-rater reliability and preliminary validity of the observation instrument are investigated.

## Methods

### Participants and procedure

At first, all infants, toddlers and (pre)school children (aged 0–7 years) treated for T1DM and their parents were recruited from Kidz&Ko, a partnership between seven pediatric diabetes clinics, and Diabeter, a national center for pediatric and adolescent diabetes care and research. Due to a small sample size, we also recruited all children (0–7 years) with T1DM from 7 other hospitals in the Netherlands (Isala Clinics Zwolle, Amphia Hospital Breda, Franciscus Hospital Roosendaal, Academic Hospital Maastricht, Medical Spectrum Twente Enschede, Zorg Groep Twente Almelo/Hengelo, Atrium Medical Center Heerlen). In these 15 hospitals, 138 young children with T1DM were treated. Parents who lacked basic proficiency in Dutch were excluded, as well as children who were mentally disabled and/or had Down syndrome, or were diagnosed with an Autism Spectrum Disorder (total families excluded: n = 17). Of the 121 eligible parents of children with T1DM, 77 families (64%) agreed to participate. Reasons for not participating were: not willing to be videotaped (n = 18), a recent hospitalization of the child (n = 3), loss of a family member (n = 1) or personal reasons (n = 22).

During a home-visit, a diabetes-specific situation (i.e., mealtime observation) and a free play situation were videotaped. During the structured 10-minute free play situation (playing with clay or making a puzzle) one of the parents and their child were asked to play together as they normally would do (this parent was also the focus of the parent–child interaction during the diabetes-specific situation). Furthermore, parents were asked to fill out a questionnaire with (socio) demographic characteristics (i.e., gender of the child, age of the child, marital status parents, and educational levels of both parents) and clinical characteristics (i.e., treatment regimen, times they monitored their child’s blood glucose level a day (average), and years since diagnosis), specifically designed for this study. Glycosylated hemoglobin (HbA_1c_), measured closest to the home-visit, was locally determined at the hospital the child was treated and extracted from the medical record. Furthermore, to examine preliminary validity of the observation instrument for diabetes-specific situations, we asked the parents about their children’s behavior by filling out the Strengths and Difficulties Questionnaire (SDQ) [25]. When examining the preliminary validity of the observation instrument, we only used the data of SDQ questionnaires of the parent that was the focus during the videotaped situation. The SDQ is a brief behavioral screening questionnaire and measures the presence of psychosocial problems and the strengths of the child. The questionnaire consists of 25 items, covering the following five domains: emotional problems, conduct problems, hyperactivity/inattention, peer relationship problems, and pro-social behavior. The 25 items are formulated on the basis of propositions and relate to the past 6 months. Some propositions are oppositely formulated. Therefore, the subscales have a bipolar character: a low score not only means that there are few problems, but also that there are strengths [25]. Research showed that the Dutch translation of the SDQ has acceptable to good psychometric properties [26].

The study was approved by the Medical Ethical Review board of St. Elisabeth Hospital Tilburg (date: 25-05-2010) and in conjunction with the Helsinki Declaration on human research.

### Constructing the observational rating scale

**Figure 1:**
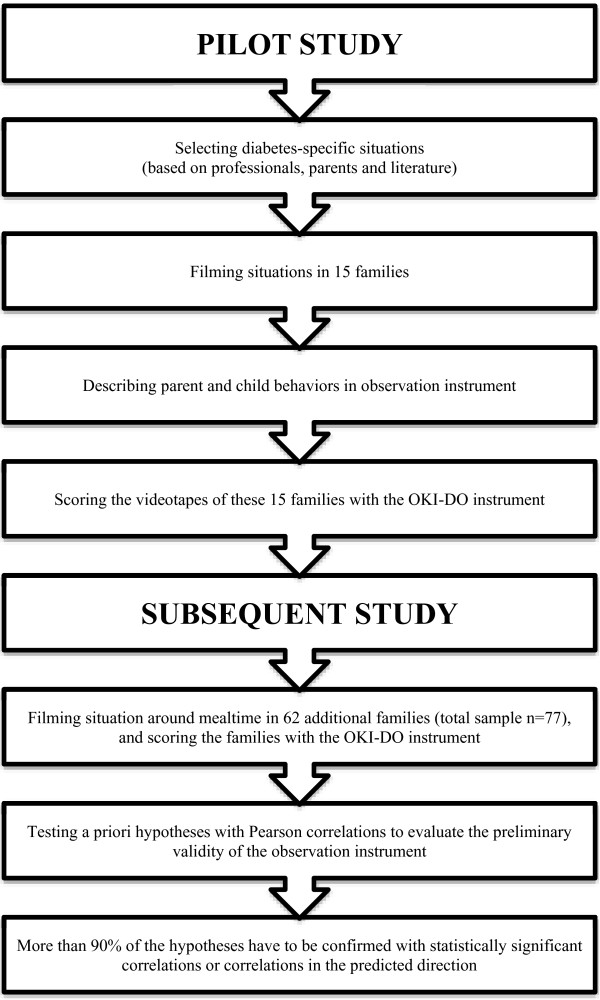
Constructing the OKI-DO observation instrument.

The development of the observation instrument has proceeded in (1) a pilot study in which we developed and evaluated the observation instrument, and (2) a subsequent study to assess preliminary validity of the observation instrument for diabetes-specific situations [3].1. In the pilot study (n = 15) we developed the observation instrument in four steps (see Figure 1):

The first step was to determine diabetes-specific situations that were most salient and/or problematic for parents and children with T1DM. The selection of these situations was based on literature and interviews with four pediatricians, four diabetes-nurses and four randomly selected parents (parents who were in the waiting room after our visit to the pediatrician) in which they were asked: “*In which situations parents might encounter problems with the diabetes-management and behavior of their child?”.*

In the second step we videotaped the most salient diabetes-specific situation and a generic situation (free play) during a two-hour home-visit in 15 families after receiving written consent from the parents. The observer did not participate in the family interactions during the diabetes-specific situation and free play situation. Because the observer did not participate and kept herself aloof from the situation, no observer effects are expected. The behaviors recorded during the free play situations were scored with an observation instrument to assess generic parent–child interaction, developed by Erickson, Sroufe and Egeland [24]. This generic observation instrument consist of six parent domains (“Supportive presence”, “Respect for child’s autonomy”, “Structure and limit setting”, “Quality of instruction”, “Hostility”, and “Confidence”), and eight child domains (“Negativity”, “Avoidance of parent”, “Compliance/child complies with parent’s task direction”, “Affection toward parent”, “Persistence”, “Reliance on parent for help”, “Enthusiasm”, and “Experience of the session”). At the end of each home-visit, parents were asked if the videotaped situations were typical or different from other days (for example, if they and/or their children did behave more active or more withdrawn than they normally do).

The third step was to describe all videotaped behaviors in an observation instrument for diabetes-specific situations (based on generic observation instruments [22-24], but specifically described to assess the quality of parent–child interaction during diabetes-specific situations).

The fourth and final step of the pilot study was to test the usability and the inter-rater reliability of the developed observation instrument. Results of the pilot study were used to refine the observation instrument for diabetes-specific situations.

2. Second, we conducted the same home-visit as in the pilot study in 62 additional families (total sample n = 77), to collect data for preliminary validity of the observation instrument.

### Statistical analyses

In the pilot study, the usability of the observation instrument was determined by a debriefing questionnaire in which the raters and authors were asked to appraise the usability of the observation instrument (Were the instructions clear?; Did instructions need further explications?; Were the descriptions of behaviors complete?; Were all behaviors covered?). To determine the inter-rater reliability [27], two raters (HvB and AN), with previous experience in observing and rating behaviors with generic observation instruments, scored the first 15 videotapes independently. A weighted kappa between 0.61-0.80 is generally regarded as an indication of substantial agreement [27].

The validity of observational rating scales is often assumed and not examined [28], however, we did investigate preliminary validity of the observation instrument in a subsequent study. The preliminary validity was evaluated by testing a priori hypotheses on the association between the observation instrument for diabetes-specific situations (observation during a diabetes-specific situation) and an observation instrument to assess generic parent–child interaction [24] (observation during free play). The hypotheses were based on the model of Belsky [29], which encompasses three determinants that influence parenting: characteristics of the parent, characteristics of the child, and contextual sources of stress (in this case T1DM) and support. For example: children, who show lots of affection toward their parent, probably contribute to a better quality of parent–child interaction. Or parents, who are hostile toward their child, probably contribute to a lower quality of parent–child interaction. Also, as youth (11–18 years) with T1DM with a disturbed parent–child relationship show more behavioral problems [6], a priori hypotheses between the domains of the observation instrument for diabetes-specific situations and SDQ subscales or total score were formulated to further examine the preliminary validity of the observation instrument. At least 75% of the hypotheses must be confirmed by a correlation (significant or non-significant trend) in order to demonstrate the preliminary validity of the observation instrument [30]. Furthermore, effects sizes will also be examined. According to Cohen, r of 0.1, 0.3, and 0.5 can be considered as small, medium and large effects, respectively [31].

## Results

### Participants

Table 1 summarizes the characteristics of participating parents and children. Among the participating children, there were 41 boys (53%). The children with T1DM had a mean age of 5.12 years (SD = 1.52, range: 2–7 years). Most children (82%) received pump therapy. On average, parents monitored their child’s blood glucose 6 times a day (range: 2–20). The mean HbA_1c_ value of the children was 59 mmol/mol or 7.6% (range 32–80 mmol/mol or 5.1% - 9.5%). Of the 74 mothers and 3 fathers that were observed, 67 mothers (91%) and 3 fathers (100%) completed the form with the (socio) demographic characteristics and SDQ [25]. Most mothers (83%) and fathers (100%) were cohabiting or married/registered partners (7% of the mothers and 0% of the fathers were single). Half of the participating mothers (50%) had a higher educational level (i.e., approximately 12 years of formal education), while all fathers (100%) had a Bachelor’s or Master’s degree (i.e., approximately 15 years of formal education).

**Table 1:** Sociodemographic and clinical characteristics of young children with type 1 diabetes and their parents

			**N (%)**	**M**	**SD**
**Children**	Sex	Boys	41 (53%)		
		Girls	36 (47%)		
	Age (years)	(range 2-7)		5.12	1.52
	HbA_1c_	(range 32 mmol/mol-80 mmol/mol)	77	59.25	9.03
		(range 5,1% - 9,5%)	77	7.6%	0.8%
	Treatment	Insulin pump	63 (82%)		
		Multiple daily insulin injections	14 (18%)		
	Blood glucose monitoring	Times a day (range 2-20)		6.08	2.69
	Years since diagnose	(range 1-6 years)		2.61	1.44
**Parents**	Total	Mothers observed	74 (96%)		
		Fathers observed	3 (4%)		
	Marital status (mothers)	Single	5 (7%)		
		Cohabiting with partner	10 (13%)		
		Married/registered partners	52 (70%)		
		Missing	7 (10%)		
	Marital status (fathers)	Single	0 (0%)		
		Cohabiting with partner	1 (33%)		
		Married/registered partners	2 (67%)		
		Missing	0 (0%)		
	Educational level (mothers)	Primary education	1 (1%)		
		12 years of formal education	36 (49%)		
		15-16 years of formal education	29 (39%)		
		Other	1 (1%)		
		Missing	7 (10%)		
	Educational level (fathers)	Primary education	0 (0%)		
		12 years of formal education	0 (0%)		
		15-16 years of formal education	3 (100%)		
		Other	0 (0%)		
		Missing	0 (0%)		

### Pilot study

When interviewing the experts about which situation to observe, glucose monitoring and the mealtime were both mentioned ten times. Nighttime (because of possible nocturnal hypoglycemia), unexpected situations (such as unexpected treats) and diabetes-management at school were mentioned nine, seven and one time(s) respectively. These situations were confirmed by the literature [11,15-18,32-41]. Because unexpected daily situations, nighttime observations, and school observations are of course more difficult and impractical to record, and filming the night-time situation can be perceived as too intrusive, we refrained from using these situations in our study. Therefore, we decided to observe the mealtime situation (including glucose monitoring and insulin administration) as this was also most frequently mentioned by the experts. Because of work, daycare and/or school, we videotaped dinnertime with all siblings and both parents present (only one parent was observed for scoring). If it was not possible to observe dinnertime, we videotaped lunchtime (with siblings, but only one parent present). On average, the mealtime lasted about 25 minutes.

After videotaping the mealtime situation (including glucose monitoring and insulin administration) in 15 families, parents of two families indicated that they or their children acted a bit different in the beginning of the home-visit when the camera was introduced (before the actual observation started), but also that after a while they themselves and their children did not even notice the camera. Based on the videotapes, the observer described all observed parent behaviors (e.g., being emotionally involved) and child behaviors (e.g., crying or accepting) in the observation instrument for diabetes-specific situations (based on generic observation instruments [22-24], but with a focus on the parent–child interaction related to the child’s diabetes and diabetes-related tasks). The observed behaviors, together with expert views of two other observers, a pediatrician and a diabetes nurse, and generic domains of parent–child interaction [22-24], resulted in a qualitative observation instrument for scoring behavior during mealtime (including glucose monitoring and insulin administration) in young children with T1DM. The observation instrument was named *OKI-DO* (OKI-DO, which literally means **O**uder **K**ind **I**nteractie-**D**iabetes **O**nderzoek: Parent Child Interaction-Diabetes Research). The qualitative observation instrument comprises ten domains to assess the quality of the parent–child interaction in diabetes-specific situations, including four parent domains (“emotional involvement”, “limit setting”, “respect for autonomy”, and “quality of instruction”), four child domains (“negative behavior”, “avoidance”, “cooperative behavior”, and “child’s response to injection”), and two family-domains (“emphasis on diabetes” and “mealtime structure”). All the domains consist of qualitative descriptions of the behavior or situation on a 5-point Likert scale. Higher scores reflect more of the behavior (e.g., a high score on ‘emotional involvement’ means the parent is highly emotional involved, and a high score on ‘negative behavior’ means the child shows a lot of negative behavior). An example of the domain “respect for autonomy” can be rated varying from score 1: *‘The caregiver receives this score if he/she fully determines what should happen without explaining anything to the child and with a visible lack of respect for the autonomy. For example: the caregiver just takes the finger of the child to check the glucose, (harshly) ‘pulls’ the child in the correct position to operate the insulin pump or determines (without consulting or warning the child) where and when the insulin injection takes place, the caregiver fully determines what and how much the child eats. If the child is (rather) independent in managing his/her diabetes, the caregiver receives this score if he/she repeatedly interferes when the child is managing his/her diabetes, while it is clear from the observation that the child can perform everything on its own’,* to score 5: *‘The caregiver receives this score if he/she praises initiatives of the child and encourages the child to make decisions on his own regarding his/her diabetes. The child may, for example, read the glucose meter, operate the insulin pump or determine where and when the insulin injection takes place (the caregiver could of course check the things his/her child does, but is herein not at all intrusive). Everything is determined in consultation with the child and the child is treated with respect.’* The parent–child dyad will receive one score (1–5) on all ten domains of the OKI-DO instrument. All domains, like “respect for autonomy” as described above, focus on the parent–child interaction related to the child’s diabetes and diabetes-related tasks. Finally, we have written a manual with detailed instructions how to videotape, observe and score the quality of the parent–child interactions.

The last step of the pilot study was to test the usability and the inter-rater reliability of the concept version of the OKI-DO instrument. The responses on the debriefing questionnaire (see *Statistical analyses*) to determine the usability, yielded a few minor improvements. These improvements consisted of specifying the instructions and more detailed descriptions of specific behaviors. Two raters (HvB and AN) with experience in rating with generic observation instruments independently scored the videotapes of the 15 families with the improved OKI-DO instrument. Weighted kappa was 0.73, indicating a good inter-rater reliability [27].

To conclude, the pilot study resulted in an observation instrument that appeared to be usable and reliable to assess parent–child interaction during mealtime (including glucose monitoring and insulin administration) in families with a young child with T1DM.

### Subsequent study

In the subsequent study, we scored all families with the OKI-DO instrument (total n = 77). Table 2 shows the mean, minimum and maximum scores on the domains of the OKI-DO instrument. As Table 2 shows, the current sample consists of rather high-functioning families (low scores on ‘*negative behavior’*, ‘*avoidance’*, ‘*response to injection’*, and high scores on ‘*limit setting*’, ‘*respect for autonomy’*, ‘*cooperative behavior’*, which means that the participating families in our study did not encounter major problems during mealtime, glucose monitoring and insulin administration).

**Table 2:** Mean, minimum and maximum scores on the OKI-DO domains

**OKI-DO domains**	**Mean**	**Minimum**	**Maximum**
Emotional involvement	3,9	2	5
Limit setting	4,2	2	5
Respect for autonomy	4,1	2	5
Quality of instruction	3,5	1	5
Negative behavior	1,5	1	4
Avoidance	1,7	1	4
Cooperative behavior	4,1	2	5
Child’s response to injection	1,7	1	4
Emphasis on diabetes	2,5	1	5
Mealtime structure	3,6	1	5

To investigate preliminary validity of the OKI-DO instrument, we tested Pearson correlation coefficients between the OKI-DO instrument (observations during mealtime, including glucose monitoring and insulin administration) and a generic observation instrument [24] (observation during free play). Also correlations between the OKI-DO instrument and SDQ subscales or total score were examined to further examine the preliminary validity of the OKI-DO instrument. Table 3 (boldface) and Table 4 show the a priori hypotheses of predicted correlations between the OKI-DO instrument and generic observation instrument to assess generic parent–child interaction [24] or the SDQ [26].

**Table 3:** **Correlation coefficients between the OKI-DO instrument and generic observation instrument**[24]**during free play**

**Free play (generic)**	**OKI-DO**	***Parent***				***Child***				***Family***	
		**Emotional involvement**	**Limit setting**	**Respect for autonomy**	**Quality of instruction**	**Negative behavior**	**Avoidance**	**Cooperative behavior**	**Child’s response to injection**	**Emphasis on diabetes**	**Mealtime structure**
*Parent’s*	Supportive presence	.38^**^	.22	.24^*^	.02	-.13	**-.15**	.16	.14	-.13	.16
	Respect for child’s autonomy	.40^**^	.15	.31^**^	.20	-.12	**-.22**	.17	.03	-.01	.00
	Structure and limit setting	.18	.31^**^	.23	**.05**	**-.20**	-.14	**.20**	.01	-.10	.24^*^
	Quality of instruction	.17	.12	.09	**.07**	-.13	.01	.00	.03	-.05	.03
	Hostility	-.35^**^	-.11	-.45^**^	-.08	-.02	.28^*^	-.18	-.12	.16	-.02
	Confidence	.18	.35^**^	.14	-.04	-.28^*^	-.14	**.23**	.02	-.13	.38^**^
*Child’s*	Negativity	**-.19**	-.15	-.16	**-.12**	**.14**	.08	**-.14**	-.10	.13	.01
	Avoidance of parent	-.09	-.13	-.19	-.08	.12	.30^**^	-.01	-.01	.08	-.02
	Compliance	.18	.26^*^	.33^**^	**.21**	**-.10**	-.15	**.14**	.14	-.09	.05
	Affection toward parent	.28^*^	.18	.33^**^	.07	-.17	**-.13**	.18	.16	-.03	.01
	Persistence	.16	.28^*^	.01	-.03	-.09	-.04	.19	.09	.22	.26^*^
	Reliance on parent for help	.15	-.09	-.10	-.00	.04	.07	-.07	.07	.02	.02
	Enthusiasm	.14	.12	.08	-.17	-.01	.02	.04	.18	.27^*^	-.06
	Experience of the session	.33^**^	.25^*^	.29^*^	.02	-.05	-.00	.12	.19	.07	.18

**.xx** = Expected correlation.

*Correlation is significant on a 0.05 level.

**Correlation is significant on a 0.01 level.

**Table 4:** **Correlation coefficients between the OKI-DO observation instrument and SDQ**[26]**subscales or total score**

**OKI-DO instrument**	**Expected direction:**	**SDQ scale:**	**Result:**
Emotional involvement	+	Total problems	.10
Limit setting	-	Conduct problems	-.23
Respect for autonomy	+	Prosocial behavior	.11
Quality of instruction	-	Total problems	-.14
Negative behavior	+	Conduct problems	.20
Avoidance	-	Prosocial behavior	-.19
Cooperative behavior	-	Conduct problems	-.27^*^
Child’s response to injection	xxx	xxx	xxx
Emphasis on diabetes	xxx	xxx	xxx
Mealtime structure	-	Conduct problems	- .11

*Correlation is significant on a 0.05 level.

Table 3 shows the correlation coefficients between the OKI-DO instrument during mealtime (including glucose monitoring and insulin administration) and the generic observation instrument [24] during free play. As Table 3 shows, 32 out of 34 (94%) hypothesized correlations (boldface) between the OKI-DO instrument and the generic observation instrument [24] showed small to medium effect sizes [31] and were confirmed with 19 statistically significant correlations (range |0.24| to |0.45|) and 13 non-significant trends (range |0.10| to |0.23|, as a correlation of 0.24 was significant, we decided that correlations of |0.10| or higher were non-significant trends). This distribution is a positive indication for the preliminary validity of the OKI-DO instrument. The OKI-DO domain “quality of instruction”, showed a zero correlation with the generic domains “structure and limit setting”, and “quality of instruction”, although we expected a (significant) correlation.

Furthermore, we found a few unpredicted significant correlations between the OKI-DO domains and generic domains [24]. However, these correlations are no evidence for or against the preliminary validity.

Table 4 shows the correlation coefficients between the OKI-DO instrument and the SDQ [25] subscales or total score. All (100%) of the hypothesized correlations showed small to medium effect sizes [31] and were confirmed with statistically significant correlations or non-significant trends. This is a further positive indication for the preliminary validity of the OKI-DO instrument.

To conclude, the present study showed encouraging indications for the usability, inter-rater reliability, and preliminary validity of the OKI-DO observation instrument to assess parent–child interaction in young children with T1DM during mealtime (including glucose monitoring and insulin administration).

## Discussion

The purpose of the present study was to develop a qualitative observation instrument to assess parent–child interaction in young children (0–7 years) with T1DM in diabetes-specific situations. In a pilot study (n = 15) we developed the OKI-DO observation instrument for scoring parent and child behavior during mealtime (including glucose monitoring and insulin administration), which consists of: “emotional involvement”, “limit setting”, “respect for autonomy”, “quality of instruction”, “negative behavior”, “avoidance”, “cooperative behavior”, “child’s response to injection”, “emphasis on diabetes”, and “mealtime structure”. The OKI-DO instrument appeared to be suitable to assess parent–child interaction in diabetes-specific situations, as weighted kappa indicated a good inter-rater reliability and the subsequent study showed positive indications for the preliminary validity. We examined the preliminary validity of our instrument in the total sample (n = 77) by testing hypothesized correlations between the OKI-DO instrument, a generic observation instrument [24] and psychosocial characteristics of the child (SDQ) [25]. We investigated multiple associations but decided against using, for example, Bonferroni correction. However, as recommended by Nakagawa [42], we examined the effect sizes and found that almost all hypothesized correlations showed a small to medium effect size [31]. Furthermore, more than 90% of the hypothesized correlations were statistically significant or showed promising but not significant trend. The correlations between the SDQ questionnaire and the OKI-DO domains almost all showed non-significant trends. This could be due to the fact that the hypotheses were based on research with youth (11–18 years) with T1DM [6]. The OKI-DO domain “quality of instruction”, however, showed some zero correlations with some of the generic domains, although we expected a (significant) correlation. The zero correlations between the OKI-DO domain “quality of instruction” and the generic domains “quality of instruction” and “structure and limit setting” may be explained by the different instructions that parents give when they are playing with their child compared to the instructions given during a medical procedure (like glucose monitoring). In this latter case, more assistance or a specific order in instructions may be required. This may have affected the results on this OKI-DO domain. Because the differences between instructions for play or during a medical procedure, we decided to keep the OKI-DO domain “quality of instruction”.

Our sample size was lower than anticipated. Unfortunately, we were not able to include the 120 families we aimed to include [3]. Despite a participation rate of 64% (70% was expected), we included 77 families (this is approximately 10% of the total population of children with T1DM aged 0–7 years in the Netherlands [43]), although 15 hospitals participated in our study instead of 7 [3]. It is possible that families with problems during mealtime, glucose monitoring and/or insulin administration were reluctant to participate in our study. However, for reasons of confidentiality, we do not have non-response data and therefore are not able to further underpin this statement. Because the families in our sample did not encounter major problems during mealtime, glucose monitoring and insulin administration (low scores on ‘*negative behavior’*, ‘*avoidance’*, ‘*response to injection’*, and high scores on ‘*limit setting’*, ‘*respect for autonomy’*, ‘*cooperative behavior’*, see Table 2), we should regard our findings as preliminary evidence supporting the validity of the OKI-DO instrument. In future research, families who encounter problems during diabetes-specific situations should definitely be included to further examine the validity of the OKI-DO instrument.

Though widely used generic observation instruments [22-24] have been developed (based on theory or observations), studies that test the validity of observation instruments are scarce [28]. The preliminary validity of the OKI-DO instrument, however, was examined in the present study and showed positive indications for the preliminary validity. In the present study, we also investigated the inter-rater reliability of the OKI-DO instrument (weighted kappa was 0.73, indicating good inter-rater reliability). To further investigate the reliability of the OKI-DO instrument, test-retest reliability could be examined in future research.

In our sample, 59.7% of the children had HbA_1c_ levels above the recommended ISPAD guideline of 58 mmol/mol or 7.5% [44]. This is in line with a recent large-scale European study [45] where 58% of the 27.035 participating children had a suboptimal HbA_1c_ level. Therefore we believe that the HbA_1c_ level is representative of other Western European children. However, most families that participated in our study were Caucasian (97%) and the majority of the children received pump therapy (82%). Therefore, we have to be cautious to generalize our findings to families with a different ethnic background and to children with multiple daily insulin injections. Furthermore, the educational level of the participants was generally higher than in the total Dutch population [46]. Approximately 32% of the adults in the Netherlands have an academic Bachelor’s or Master’s degree [46]. In this study 39% of the mothers and 100% of the fathers had an academic degree. Research shows that the educational level of parents is positively associated with the quality of parent–child interaction [47] and parenting strategies [48], so the findings of this study may be more applicable for parents with higher educational levels.

## Conclusions

As diabetes-related family behaviors seem to be established in the early years post-diagnosis [9,49], interventions should start as early as possible. The incidence of young children with T1DM is increasing [50] and early detection of problems and intervening in this young patient group is necessary. Observational research has shown that parents of children with T1DM have more parenting problems during mealtime [15,17,40] and there is a need for effective parenting strategies [14]. In future research, the OKI-DO observation instrument can be used to conduct studies that can help to determine whether parent–child interaction-patterns are associated with specific diabetes outcomes, such as glycosylated hemoglobin (HbA_1c_) and psychosocial characteristics (such as quality of life). These results can than serve as a reference to determine, for example, whether interventions to improve parent–child interaction would be a meaningful intervention for families with a young child with T1DM. Furthermore, research showed that injection distress is more common in younger children and recently diagnosed children [51]. The OKI-DO instrument could enable scientists and clinical practitioners to evaluate interventions aimed at decreasing the injection distress for both parents and children and interventions aimed at optimizing the quality of parent–child interaction in families with a young child with T1DM.

## Competing interests

The authors declare that they have no competing interests.

## Authors’ contributions

EH, HvB, and FP developed the design of the study. HJA and EvM are the study coordinators of collaborating institutions. WE assisted with the statistics. AN carried out the home-visits and drafted the manuscript. All authors are considered as co-authors as they have significantly contributed to developing this research, obtaining the data, and writing the paper. All authors read and approved the final manuscript.

## Pre-publication history

The pre-publication history for this paper can be accessed here:

http://www.biomedcentral.com/1471-2431/14/145/prepub
